# Supplementary material for: Advancing mathematics by guiding human intuition with AI
Source: Nature. 2021 Dec 1;600(7887):70–4. doi: 10.1038/s41586-021-04086-x (PMC8636249; doi:10.1038/s41586-021-04086-x)
Supplement: Supplementary file 1 — This file contains Supplementary text, Figs. 1–6, hyperbolic knots and references. [file 41586_2021_4086_MOESM1_ESM.pdf]

nature portfolio

https://doi.org/10.1038/s41586-021-04086-x

Supplementary information

Advancing mathematics by guiding human intuition with AI

In the format provided by the
authors and unedited

SUPPLEMENTARY MATERIAL: THE SIGNATURE AND CUSP SHAPE OF HYPERBOLIC KNOTS

1. Introduction

The fundamental objects of topology are manifolds – spaces that locally look like n-dimensional coordinate space. Dimensions 3 and 4 are the most relevant from a physical point of view, and turn out to be the most difficult to understand mathematically. This is the subject of low-dimensional topology.

The theory of 3-manifolds is governed by geometry due to Thurston's celebrated Geometrisation Conjecture [9], which states that every 3-manifold can be obtained by gluing pieces that each have one of eight standard geometric structures, of which hyperbolic is the most common. This was proven by Perelman – a result named "breakthrough of the year" by Science in 2006.

Much of the complication in the study of 3- and 4-manifolds comes from the phenomenon of knotting, where a knot is an embedding of the circle in 3-space. A hyperbolic knot is one whose complement carries a hyperbolic geometry, and is hence a common building block of 3-manifolds. On the other hand, the classification of topological 4-manifolds is mostly determined by their algebraic invariants due to the work of Freedman [4], which earned him a Fields medal.

One of the most fundamental 4-manifold invariants is the signature. Signature can also be defined for a knot, which gives information on the complexity of surfaces that the knot can bound in the four-ball. The geometric theory of 3-manifolds and the algebraic theory of four-manifolds have so far developed with very little interaction with each other. To bridge this gap, we used techniques from machine learning combined with mathematical insights, and found that a completely unexpected relationship exists between the geometric world of 3-manifolds and the algebraic theory of 4-manifolds. We showed that a new geometric quantity called the natural slope, which describes the shape of the boundary of a hyperbolic knot complement, differs from twice the knot signature by at most a constant times the hyperbolic volume divided by the cube of the injectivity radius. This could lead to further links between two currently orthogonal areas.

2. Background on knot theory

As outlined in the introduction, knot theory plays a fundamental role in low-dimensional topology. Some of knot theory's main goals are to classify knots, to understand their properties, and to establish connections with other fields. One of the principal ways that this is done is to define invariants, which are geometric, algebraic, or numerical quantities that are the same for any two equivalent knots.

Hyperbolic invariants are those derived from hyperbolic geometry, and relate to the complement of the knot, in other words, the manifold that one obtains by removing the knot K from 3-dimensional space. One of the major milestones in the field was Thurston's introduction of the Geometrisation Conjecture and his proof

of this for knot complements [9]. This established that the complement of every knot (with an explicit list of exceptions) has a unique hyperbolic structure. In other words, this complement has a complete Riemannian metric of constant curvature -1. Associated with this metric are various natural geometric quantities, such as volume, which we denote by $\mathrm{vol}(K)$. See Figure 1 for a visualisation of the hyperbolic metric on the complement of the knot 8_5.

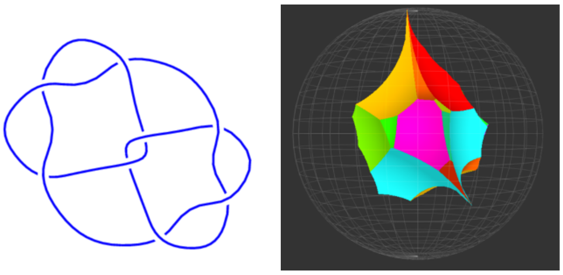

FIGURE 1. Left: The knot 8_5. Right: A fundamental domain for its hyperbolic structure, viewed as a subset of the Poincaré ball model, as created by the program SnapPy [3].

Algebraic invariants include some of the oldest invariants in knot theory, such as the Alexander polynomial defined by Alexander [1] in 1928, the fundamental group, first studied by Poincaré [8] in the late 19th century, and the signature, defined by Trotter [10] in 1962. It is the signature $\sigma(K)$ of a knot K that we will focus on here. It is a basic invariant that is extracted from a matrix associated with K, known as its Seifert matrix. It is just the number of positive eigenvalues of this matrix minus the number of negative eigenvalues. It controls many key properties of the knot, including its 4-dimensional behaviour. One particularly fruitful area of research in knot theory is to consider $\mathbb{R}^3$ as being the boundary of upper half space $\mathbb{R}^4_+ = \{(x_1, x_2, x_3, x_4) : x_4 \geq 0\}$ and then to investigate the surfaces that a knot K can bound in $\mathbb{R}^4_+$. Surprisingly, a non-trivial knot K can bound a (topologically locally-flat) embedded disc in $\mathbb{R}^4_+$; it is then known as 'slice.' The use of slice knots is one of the main tools in 4-dimensional topology, notably in Freedman's proof of the 4-dimensional Poincaré conjecture [4]. One of the many uses of knot signature is that it provides an obstruction to a knot being slice: any slice knot must have zero signature [7].

These two types of invariants, hyperbolic and algebraic, are derived from quite different mathematical disciplines, and so it is of considerable interest to establish connections between them. A notable example of such a potential connection is the Volume Conjecture [6], which proposes that the hyperbolic volume of a knot (a geometric invariant) should be encoded within the asymptotic behaviour of its coloured Jones polynomials (which are algebraic invariants).

3. The natural slope of a hyperbolic knot

In our work, we have found a new and surprising connection between a hyperbolic invariant, which we call 'natural slope,' and the signature. The natural slope is a quantity extracted from the maximal cusp, which is the part of the knot complement near the knot itself. Specifically, the hyperbolic structure specifies a Euclidean

metric on the torus that encloses the knot. From this metric, the meridian and longitude of the knot determine translations of the Euclidean plane, which can be specified by complex numbers λ and μ. These numbers are readily computed using the program SnapPy [3]. See Figures 3 and 4 for illustrations for the figure eight knot and the knot 12a52.

Our analysis using machine learning strongly suggested that there is a relationship between meridional translation μ, the longitudinal translation λ and signature $\sigma(K)$. This relationship is best understood by means of the following new quantity. The 'natural slope' is defined to be $\mathrm{slope}(K) = \mathrm{Re}(\lambda/\mu)$, where Re denotes the real part. It has the following geometric interpretation. One can realise the meridian curve as a geodesic γ on the Euclidean torus. If one fires off a geodesic $\gamma^{\perp}$ from this orthogonally, it is will eventually return and hit γ at some point. In doing so, it will have travelled along a longitude minus some multiple of the meridian. This multiple is the natural slope. It need not be an integer, because the endpoint of $\gamma^{\perp}$ might not be the same as its starting point. See Figure 2 for an illustration.

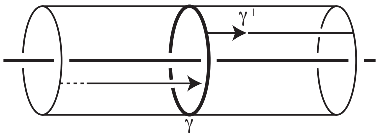

Figure 2. A schematic picture of the cusp torus. A geodesic is shown running in the direction $\gamma^{\perp}$ that is perpendicular to the meridian γ. By the time it returns to γ, it has travelled one longitude minus some multiple s of the meridian. This real number s is the natural slope of K.

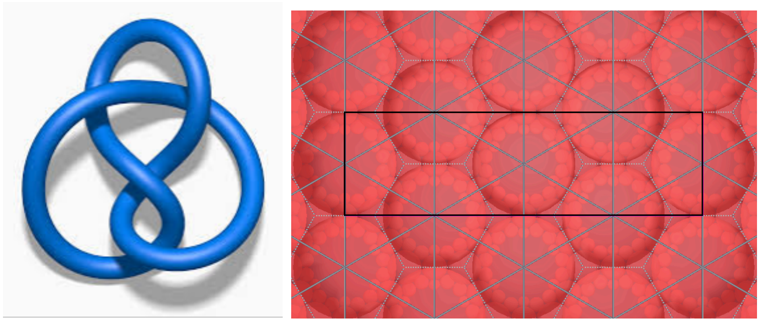

Figure 3. Left: The figure-eight knot 4_1. Right: A picture of its cusp torus, which is obtained by identifying opposite sides of the rectangle. The horizontal and vertical sides of this rectangle give $\lambda = 3.4641$ and $\mu = i$. The natural slope is 0 and the signature is 0. The diagram of the cusp torus was produced using SnapPy [3].

Our initial conjecture relating natural slope and signature was as follows.

Conjecture 3.1. *There exist constants c_1 and c_2 such that, for every hyperbolic knot K,*

$$|2\sigma(K) - \mathrm{slope}(K)| < c_1 \,\mathrm{vol}(K) + c_2.$$

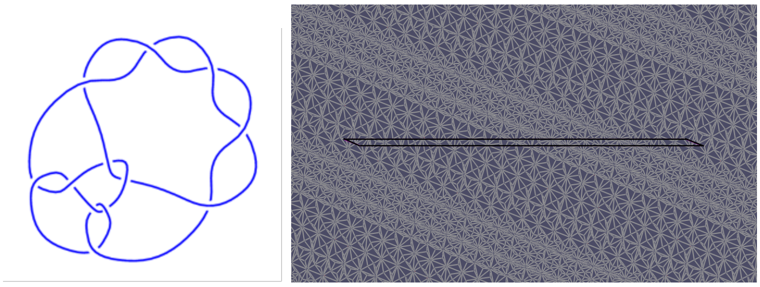

FIGURE 4. Left: The knot 12a52. Right: Its cusp torus. The longitude and meridian are 27.7228 and $-1.2838 + 0.5145i$. Its natural slope is -18.6064 and its signature is -8. Note how far the parallelogram is from being right-angled; this is the defining feature of having very positive or very negative slope.

This conjecture was strongly supported by an analysis of large datasets, including the knots up to 16 crossings in the Regina dataset [2] and a dataset of knot constructed using `random_link` in SnapPy, having 10 to 80 crossings in their SnapPy-simplified form (see Figure 5).

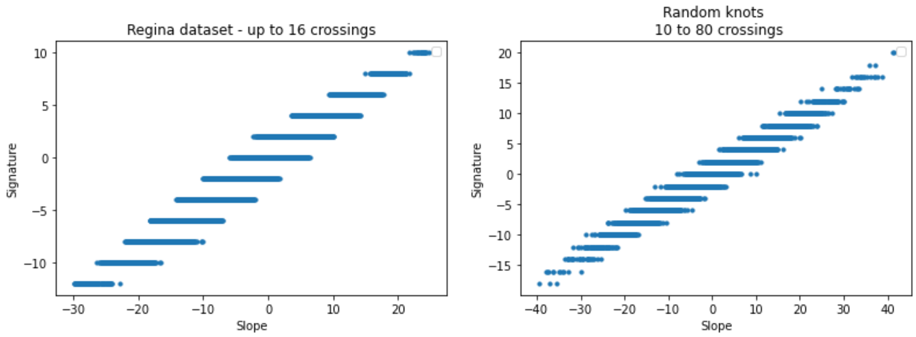

FIGURE 5. Scatter plots of signature $\sigma(K)$ against slope(K). Left: The knots up to 16 crossings in the Regina dataset. Right: Knots randomly sampled using the command `random_link` in SnapPy, having 10 to 80 crossings in their SnapPy-simplified form.

However, counterexamples to this conjecture were eventually found using braids. Specifically, we considered a sequence of knots K_n, which are obtained as closures of the braid $\sigma_1\sigma_2^{-1}(\sigma_1\sigma_2)^{3n}$ (see Figure 6). Their signatures are equal to $-4n$. Their slopes satisfy $\text{slope}(K_n)/(-9n) \to 1$ as n tends to infinity. Therefore, the quantity $|2\sigma(K_n) - \text{slope}(K_n)|$ is unbounded as n tends to infinity. On the other hand, their volumes are bounded above by 4.0598. Hence, there cannot be constants c_1 and c_2 as in the conjecture.

4. Main theorem

Even though our conjecture turned out to be false in the form initially stated, we were able to establish a relationship between slope(K), signature $\sigma(K)$, volume

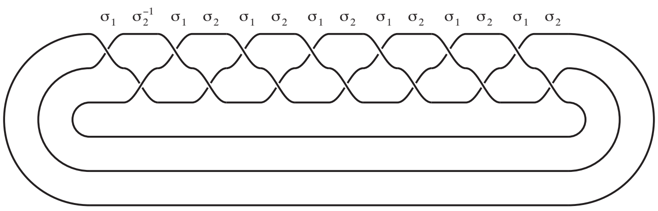

FIGURE 6. The knot K_n in the case $n = 2$. It has signature -8 and slope -18.2151. Its volume is 2.8281.

vol(K) and another important geometric invariant, the injectivity radius inj(K), which is defined as follows.

For a Riemannian manifold M, its injectivity radius at a point p is defined to be the supremal value of r with the property that the exponential map based at p is injective when restricted to a ball of radius r. It is denoted $\mathrm{inj}_p(M)$. The injectivity radius of M is defined to the infimum of $\mathrm{inj}_p(M)$, over all points p in M. When the manifold is closed and hyperbolic, this is just half the length of the shortest geodesic. However, the manifolds we are considering are knot complements, and so are not closed, and their injectivity radius, with its usual definition, is zero. In our case, we instead define inj(K) to be the infimal value of $\mathrm{inj}_p(M)$, over all points p outside of the maximal cusp. This can readily be computed using SnapPy, as it is a simple function of the length of the shortest geodesic and the length of the shortest slope on the boundary of the maximal cusp.

Our main theorem is as follows.

Theorem 4.1. *There exists a constant c_3 such that, for any hyperbolic knot K,*

$$|2\sigma(K) - \mathrm{slope}(K)| \leq c_3 \ \mathrm{vol}(K)\, \mathrm{inj}(K)^{-3}.$$

It turns out that injectivity radius tends not to get very small, even for manifolds of large volume (see Figure 7). Hence, the term $\mathrm{inj}(K)^{-3}$ tends not to get too large in practice. However, it would clearly be desirable to have a theorem that avoided the dependence on $\mathrm{inj}(K)^{-3}$. We give such a result in the next section.

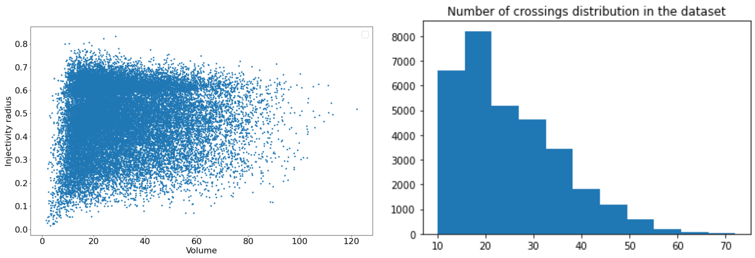

FIGURE 7. Left: A scatter plot of volume and injectivity radius for a random sample of knots. This sample was created by randomly selecting a knot diagram with at most 80 crossings, using the command `random_link` in SnapPy. Right: The distribution of crossings in the knot diagrams in this dataset.

5. A refined theorem

Although Conjecture 3.1 turned out not to be true, it is natural to wonder whether the signature $\sigma(K)$ can be estimated in terms of geometric invariants, with an error bound depending only on the volume, and not depending on the injectivity radius. We were able to establish such a bound. It is sharper than Theorem 4.1, although not as simply stated.

Our estimate for $\sigma(K)$ is stated as $\mathrm{slope}(K)/2$ plus a correction term. This correction term involves all the 'short' geodesics. For the sake of being definite, we consider all the geodesics with length less than 1. Let OddGeo be the collection of all such geodesics that have odd linking number with the knot. For each such geodesic, we will define a 'correction term' $\kappa(\mathrm{tw}_p(\gamma), \mathrm{tw}_q(\gamma))$. Our theorem is then as follows.

Theorem 5.1. *There is a constant c_4 such that for any hyperbolic knot K, the quantities $\sigma(K)$ and*

$$\mathrm{slope}(K)/2 - \sum_{\gamma \in \mathrm{OddGeo}} \kappa(\mathrm{tw}_p(\gamma), \mathrm{tw}_q(\gamma))$$

differ by at most $c_4 \,\mathrm{vol}(K)$.

The details of the correction term are somewhat technical, but we state them here for the sake of completeness.

Definition 5.2. Let γ be a geodesic with complex length $\mathrm{cl}(\gamma)$. Here, $\mathrm{cl}(\gamma)$ is chosen so that its imaginary part lies in $(-\pi, \pi]$. The *twisting parameter* $\mathrm{tw}(\gamma)$ is the pair (p, q) of coprime integers satisfying the following:

(1) p is even, and q is odd and non-negative;
(2) subject to this condition, the quantity $|\mathrm{cl}(\gamma)p + 2\pi i q|$ is minimised;
(3) if there are several values of (p, q) for which this quantity is minimised, then choose the one that is minimal with respect to lexicographical ordering.

We denote $\mathrm{tw}(\gamma)$ by $(\mathrm{tw}_p(\gamma), \mathrm{tw}_q(\gamma))$.

Once one has the integers $\mathrm{tw}_p(\gamma)$ and $\mathrm{tw}_q(\gamma)$, the correction term is computed as follows.

Definition 5.3. For any pair of positive integers (p, q), we define the *signature correction* $\kappa(p, q)$ recursively as follows.

(1) If $p > 2q$ and q is odd, then $\kappa(p, q) = \kappa(p - 2q, q) - 1$.
(2) If $p > 2q$ and q is even, then $\kappa(p, q) = \kappa(p - 2q, q)$.
(3) If $p = 2q$, then $\kappa(p, q) = -1$.
(4) If $q \leq p < 2q$ and q is odd, then $\kappa(p, q) = -\kappa(q, 2q - p) - 1$.
(5) If $q \leq p < 2q$ and q is even, then $\kappa(p, q) = -\kappa(q, 2q - p) - 2$.
(6) If $p < q$, then $\kappa(p, q) = \kappa(q, p)$.

We extend this definition to non-zero integers p and q by defining $\kappa(-p, q) = \kappa(p, -q) = -\kappa(p, q)$. When one of p or q is zero, then $\kappa(p, q) = 0$.

It is reasonably clear that this gives a well-defined value of $\kappa(p, q)$. This is because it defines $\kappa(p, q)$ uniquely when $p = q$, and when $p \neq q$, it defines $\kappa(p, q)$ in terms of some $\kappa(p', q')$ where either $q' < q$, or $q' = q$ and $p' < p$.

Thus, Theorem 5.1 asserts that the signature of a hyperbolic knot can be computed in terms of purely hyperbolic quantities, with an error that is at most linear in the volume.

6. Applications

The relationship between the natural slope and the signature of a hyperbolic knot given in Theorems 4.1 and 5.1 has various interesting applications. On the one hand, the natural slope turns out to control the non-hyperbolic Dehn surgeries on the knot. In the other direction, the signature is known to control the genus of surfaces in $\mathbb{R}^4_+$ that the knot can bound. We now briefly describe these applications.

The *topological 4-ball genus* $g_4^{\mathrm{top}}(K)$ is the minimal possible genus of a locally-flat topologically embedded compact orientable surface in $\mathbb{R}^4_+$ with boundary equal to K. The following result provides a lower bound on $g_4^{\mathrm{top}}(K)$ in terms of purely hyperbolic data. This follows immediately from Theorem 4.1 together with the well-known inequality $g_4^{\mathrm{top}}(K) \geq |\sigma(K)|/2$ due to Murasugi [7].

Corollary 6.1. *The topological 4-ball genus $g_4^{\mathrm{top}}(K)$ of a hyperbolic knot K satisfies*

$$g_4^{\mathrm{top}}(K) \geq |\mathrm{slope}(K)|/4 - (c_3/4)\,\mathrm{vol}(K)\mathrm{inj}(K)^{-3}.$$

A significant theme in low-dimensional topology is the study of Dehn surgery. Specifically, given a knot K and fraction q/p, there is a recipe for building a 3-manifold $K(p/q)$, by drilling out a regular neighbourhood $N(K)$ of K from the 3-sphere, and then attaching a solid torus, so that the meridian of the solid torus is attached to the slope on $\partial N(K)$ that winds p times around the longitude and q times around the meridian. A major challenge in the field is to control the values of q/p for which the manifold $K(q/p)$ does not admit a hyperbolic structure [5]. The natural slope can be shown to control these values of q/p. Hence, using Theorem 4.1, the signature also constrains these values of q/p, as follows.

Corollary 6.2. *If K is a hyperbolic knot and q/p is a slope satisfying*

$$|q/p + 2\sigma(K)| > \left(6 + c_3\,\mathrm{vol}(K)\,\mathrm{inj}(K)^{-3}\right)/|p| \quad \text{or} \quad |p| > 8,$$

then the manifold $K(q/p)$ obtained by q/p Dehn surgery along K is hyperbolic.

Experimentally, the slopes q/p for which $K(q/p)$ is non-hyperbolic tend to be clustered near $-2\sigma(K)$, as the above corollary predicts. An interesting case is the $(-2,3,7)$-pretzel knot $12n242$. This has signature -8 and slope approximately -18.215. There are 7 slopes q/p for which $K(q/p)$ is not hyperbolic: 16, 17, 18, 37/2, 19 and 20. It is interesting to observe that these slopes are concentrated in a short interval $[16, 20]$ that contains both $-\mathrm{slope}(K)$ and $-2\sigma(K)$.

We expect that this newly discovered relationship between natural slope and signature will have many other applications in low-dimensional topology.

References

[1] J. W. Alexander. Topological invariants of knots and links. *Trans. Amer. Math. Soc.*, 30:275–306, 1928. [2]

[2] Benjamin A. Burton, Ryan Budney, William Pettersson, et al. Regina: Software for low-dimensional topology. `http://regina-normal.github.io/`, 1999–2021. [4]

[3] Marc Culler, Nathan M. Dunfield, Matthias Goerner, and Jeffrey R. Weeks. SnapPy, a computer program for studying the geometry and topology of 3-manifolds. Available at `http://snappy.computop.org` (08/02/2021). [2, 3]

[4] M. H. Freedman. The topology of four-dimensional manifolds. *J. Differential Geometry*, 17(3):357–453, 1982. [1, 2]

[5] C. McA. Gordon. Dehn filling: a survey. In *Knot theory (Warsaw, 1995)*, volume 42 of *Banach Center Publ.*, pages 129–144. Polish Acad. Sci. Inst. Math., Warsaw, 1998. [7]

[6] R. M. Kashaev. The hyperbolic volume of knots from the quantum dilogarithm. *Lett. Math. Phys.*, 39(3):269–275, 1997. [2]

[7] Kunio Murasugi. On a certain numerical invariant of link types. *Trans. Amer. Math. Soc.*, 117:387–422, 1965. [2, 7]

[8] Henri Poincaré. Analysis situs. *Journal de l'École Polytechnique*, 2(1):1–123, 1895. [2]

[9] William P. Thurston. Three-dimensional manifolds, Kleinian groups and hyperbolic geometry. *Bull. Amer. Math. Soc. (N.S.)*, 6(3):357–381, 1982. [1, 2]

[10] H. F. Trotter. Homology of group systems with applications to knot theory. *Ann. of Math. (2)*, 76:464–498, 1962. [2]

SUPPLEMENTARY MATERIAL: A NEW FORMULA FOR KAZHDAN-LUSZTIG POLYNOMIALS

Abstract. Kazhdan-Lusztig polynomials are important and mysterious objects in representation theory. Here we present a new formula for their computation for symmetric groups based on the Bruhat graph. Our approach suggests a solution to the combinatorial invariance conjecture for symmetric groups, a well-known conjecture formulated by Lusztig and Dyer in the 1980s.

1. Kazhdan-Lusztig polynomials and combinatorial invariance

In this background section, we give some background on Coxeter groups, Bruhat graphs, Kazhdan-Lusztig polynomials and the combinatorial invariance conjecture. Excellent references for the following include [Hum90, Bre04, BB05, Soe97, EMTW].

1.1. **Coxeter groups and Kazhdan-Lusztig polynomials.** *Coxeter groups* are an important class of groups, which arose out of H. S. M. Coxeter's study of finite reflection groups in the 1930s. They are characterised by a presentation via generators and relations. In this presentation the generating set are called the *simple reflections*. An important example of a Coxeter group is the symmetric group S_n consisting of all permutations of $0, 1, \dots, n-1$, with simple reflections consisting of the set $S = \{(i, i+1)\}$ of adjacent transpositions.

In a seminal paper [KL79], Kazhdan and Lusztig associated to any pair of elements x, y in a Coxeter group a polynomial with integer coefficients

$$x, y \in W \mapsto P_{x,y} \in \mathbb{Z}[q]$$

known as the *Kazhdan-Lusztig polynomial.* All that we say here concerning their definition is that it is highly inductive; one works one's way "out" in the group, starting at the identity and applying generators from S. At each step in the calculation one might need any of the previously computed polynomials. Thus, they are rather cumbersome to calculate by hand, but it is not difficult to compute billions of them on a computer with enough memory. (This is useful for machine learning, as one often needs access to large data sets.)

1.2. **The Bruhat graph.** To any Coxeter group one may associate its *Bruhat graph.* For the symmetric group, this is the graph with vertices corresponding to all elements of S_n, and an edge joining x and y if and only if they differ by multiplication by a transposition. (In other words, x and y are connected in the Bruhat graph if they agree on all but two elements of $0, 1, \dots, n-1$.) The symmetric group has a natural *length function* given by the number of inversions:

$$\ell(x) = \#\{i < j \mid x(i) > x(j)\}.$$

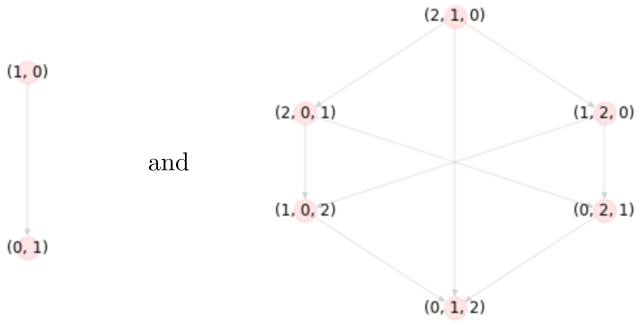

Figure 1. Ordered Bruhat graphs for $n = 2, 3$

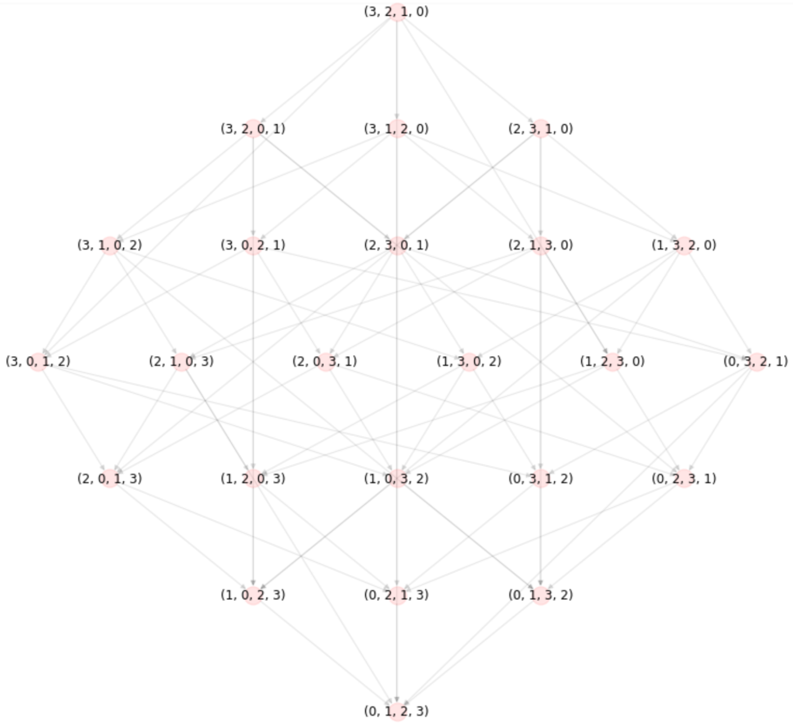

Figure 2. Ordered Bruhat graph for $n = 4$

We regard the length function as giving us a notion of "height" on the Bruhat graph. This allows us to orient the edges of the Bruhat graph via decreasing length. Figures 1 and 2 give pictures of the Bruhat graph for $n = 2, 3$ and 4.[1]

1.3. **Bruhat order.** The Bruhat graph allows us to define the *Bruhat order*, which is a partial order on W. It is defined as follows:

$$(1) \qquad x \leqslant y \quad \Leftrightarrow \quad \begin{array}{c}\text{there exists a downward path}\\ \text{from } y \text{ to } x \text{ in the Bruhat graph.}\end{array}$$

(We include paths of length zero, so $x \leqslant x$ always holds.) For example, in the symmetric group the minimal element is always the identity permutation, and the maximal element is the permutation w_0 which interchanges 0 and $n-1$, 1 and $n-2$ etc.

The Bruhat order is remarkably complex, and it has long been suspected that Kazhdan-Lusztig polynomials reflect subtle properties of the Bruhat order. An elementary manifestation of this phenomenon (easy to prove) is that:

$$P_{x,y} \neq 0 \Leftrightarrow x \leqslant y.$$

Less elementary connections tend to involve the interval $[x, y]$ consisting of the full subgraph of the Bruhat graph between x and y. (That is, this consists of all edges and vertices which may be reached from y on the way to x, whilst progressing downwards.) For example, a much less obvious fact [Car94, Dye93] is that

$$P_{x,y} = 1 \Leftrightarrow \begin{array}{c}\text{the graph obtained from } [x, y]\\ \text{by forgetting edge orientations is regular}\\ \text{(i.e. all vertices have the same degree).}\end{array}$$

(It is easy to see that the full Bruhat graph is regular, and in particular $P_{\mathrm{id},w_0} = 1$.)

1.4. **First examples.** In S_3 all proper intervals are isomorphic to the following posets[2] (as the reader may check easily, using Figure 1):

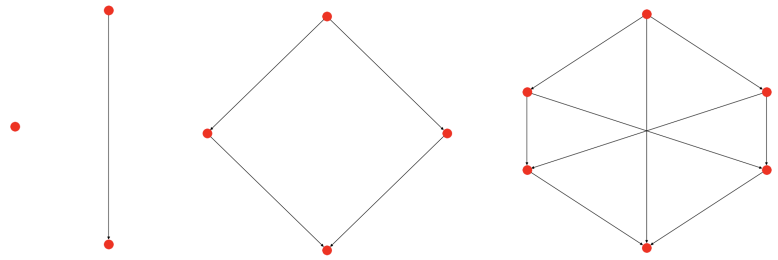

All these graphs are regular (after forgetting edge orientations), and thus all Kazhdan-Lusztig polynomials are 1.

In S_4, almost all intervals $[x, y]$ are regular, and hence almost all Kazhdan-Lusztig polynomials $P_{x,y}$ are 1. There are four intervals which are not regular. Here we depict two intervals which are not: those between 0213 and 2301, and

[1]Throughout this paper we use *string notation* for permutations. Thus $(2, 0, 3, 1)$ (or often simply 2031) denotes the permutation of $0, 1, 2$ and 3 that sends $0 \mapsto 2$, $1 \mapsto 0$, $2 \mapsto 3$ and $3 \mapsto 1$.

[2]poset = partially ordered set

between 1032 and 3120:

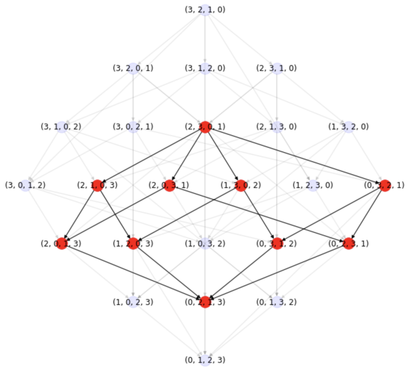

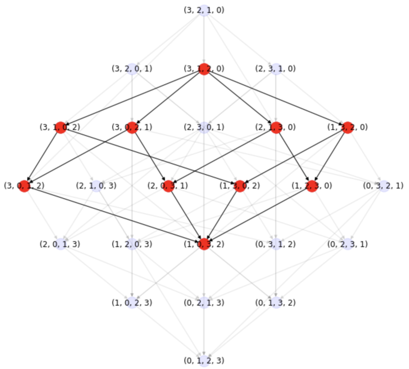

In both cases, the interval is isomorphic to the following directed graph, known as the “4 crown”:

(2)

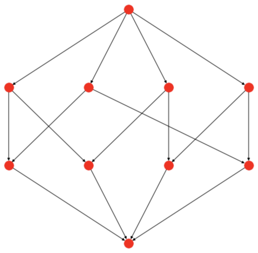

In both these cases the Kazhdan-Lusztig polynomials are equal:

$$P_{0213,2301} = P_{1032,3120} = 1 + q. \tag{3}$$

1.5. **The combinatorial invariance conjecture.** The following conjecture, formulated independently by Lusztig and Dyer in the 1980s, was a major motivation for our work on Kazhdan-Lusztig polynomials:

Conjecture 1.1. *The Kazhdan-Lusztig polynomial $P_{x,y}$ depends only on the isomorphism type of Bruhat graph of the interval $[x, y]$.*

For example, given only the ordered graph (2) (and not the labelling of its vertices) we should be able to predict the Kazhdan-Lusztig polynomial $1 + q$. The fact that (2) occurs in two diffferent ways in the Bruhat graph of S_4 with equal Kazhdan-Lusztig polynomials, can be seen as an instance of this conjecture. Figures 3 and 4 shows two more examples of the assignment of Kazhdan-Lusztig polynomial to Bruhat intervals.

Remark 1.2. The combinatorial invariance conjecture is a central conjecture in the study of Bruhat intervals. The reader is referred to [Bre04] for more detail on known cases. We do not discuss the various partial results towards the conjecture here, except to mention that it is known to hold for intervals starting at the identity [BCM06].

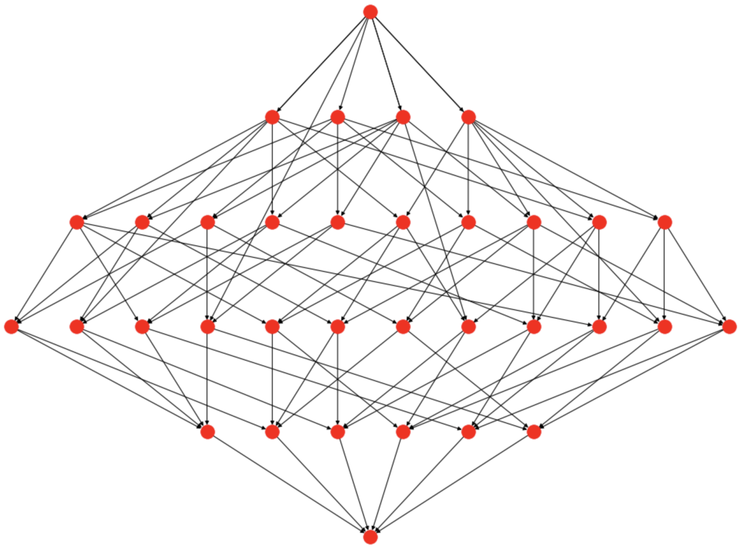

$\mapsto 1+q^2$

FIGURE 3. Interval and Kazhdan-Lusztig polynomial for x = 03214 and 34201

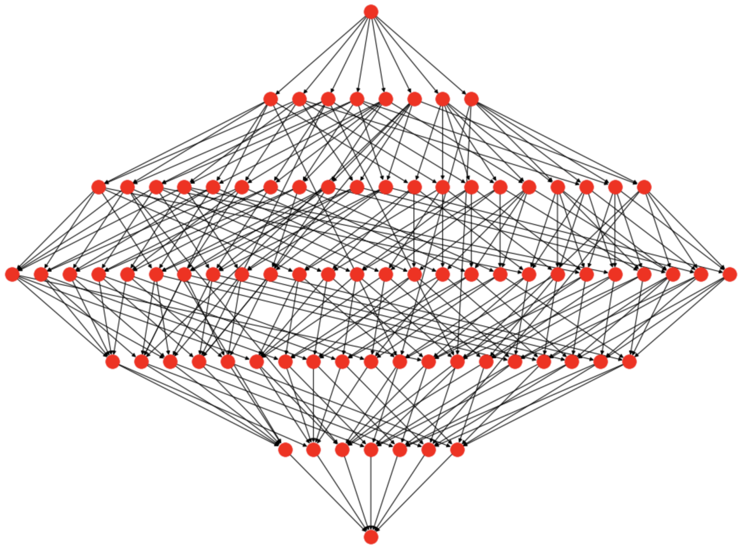

$\mapsto 1+3q+q^2$

FIGURE 4. Interval and Kazhdan-Lusztig polynomial for x = 021435 and y = 234501

2. The new formula

In this section we describe our new formula. Before going into detail, let us give a rough idea of what the formula looks like. Recall that our goal is to compute the Kazhdan-Lusztig polynomial starting from the Bruhat graph. By induction, we can assume that we can do this for any smaller graph. In particular, we can assume that all intermediate polynomials $P_{u,v}$ are known, for all $u, v \in [x, y]$ with $(u, v) \neq (x, y)$.

Our formula depends on the choice of an auxilliary structure on our graph, called a *hypercube decomposition*. Such a decomposition amounts to the choice of a subinterval $J \subset [x, y]$ satisfying certain concrete combinatorial conditions. (The reader is encouraged to skip ahead a few pages to Figure 6, where a typical hypercube decomposition is illustrated.) There always exists at least one hypercube decomposition, but in general there will be many. Any choice of hypercube decomposition determines two polynomials in q, the *inductive piece* and *hypercube piece*. Our formula is:

$$\partial P_{x,y} = \textit{inductive piece} + \textit{hypercube piece}. \tag{4}$$

The left hand side is the q-derivative of the Kazhdan-Lusztig polynomial, from which the Kazhdan-Lusztig polynomial can be recovered. The calculation of the *inductive piece* (resp. *hypercube piece*) uses only the part of the graph which lies (resp. does not lie) in J. (Again, the reader is encouraged to glance at Figure 6. The nodes necessary for the computation of the hypercube and inductive piece are in blue (resp. red).)

2.1. The q-derivative of Kazhdan-Lusztig polynomials.

We now introduce a new polynomial, whose knowledge is equivalent to knowledge of the Kazhdan-Lusztig polynomial, but which is easier to handle. Define

$$\partial P_{x,y}(q) = \frac{P_{x,y}(q) - q^{\ell(y)-\ell(x)} P_{x,y}(q^{-1})}{1-q}.$$

(One checks easily that the denominator always divides the numerator, so $\partial P_{x,y}$ is always an integer valued polynomial.) We refer to $\partial P_{x,y}$ as the *q-derivative of the Kazhdan-Lusztig polynomial*.[3] Defining properties of Kazhdan-Luztig polynomials[4] ensure that $\partial P_{x,y}$ determines $P_{x,y}$.

Example 2.1. We give three examples of Kazhdan-Lusztig polynomials, and their corresponding q-derivatives:

(1) $P_{x,y} = 1$, $\ell(y) - \ell(x) = 3$, $\partial P_{x,y} = 1 + q + q^2$, 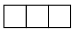 .

(2) $P_{x,y} = 1 + q$, $\ell(y) - \ell(x) = 3$, $\partial P_{x,y} = 1 + 2q + q^2$, 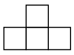 .

(3) $P_{x,y} = 1 + q^2$, $\ell(y) - \ell(x) = 6$, $\partial P_{x,y} = 1 + q + 2q^2 + 2q^3 + q^4 + q^5$, 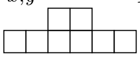 .

(We leave it up to the reader to determine the meaning of the box diagrams, as well as how to recover the Kazhdan-Lusztig polynomial from them.)

---

[3] In the related theory of Kazhdan-Lusztig-Stanley polynomials, this polynomial is often called the "H-polynomial".

[4] more precisely, the fact that their degree is bounded above by $(\ell(y) - \ell(x) - 1)/2$

2.2. **Hypercube clusters.** We work in the setting of directed acyclic graphs. Any such graph is a poset in natural way, where we declare $x \leqslant y$ if there exists a directed path from y to x.

For any finite set E, the *E-hypercube* H_E is the directed acyclic graph with:

(1) vertices consisting of subsets of E;
(2) an edge $I \to J$ if J is obtained from I by removing one element.

Example 2.2. E-hypercubes H_E for $E = \{0\}$, $\{0,1\}$ and $\{0,1,2\}$:

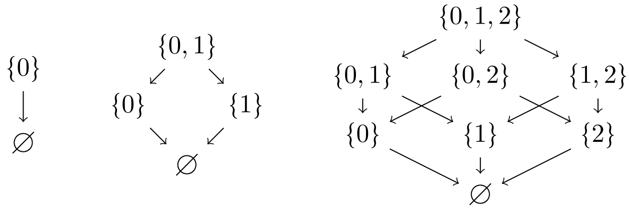

Now suppose given a directed acyclic graph X (in our applications X will be a Bruhat graph) and a node $x \in X$. We say that a subset E of edges with target x *spans a hypercube* if there exists a unique embedding (i.e. injection) of directed graphs

$$\vartheta : H_E \to X$$

sending the edge $(\{\alpha\} \to \varnothing)$ in H_E to α, for all edges α in E. If E spans a hypercube, its *crown* is $\vartheta(E)$.

Example 2.3. Consider the following directed graph (isomorphic to the Bruhat graph of S_3):

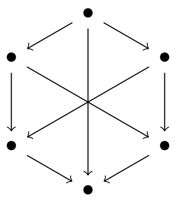

In the following we show some pairs of edges with common target with a colour. The pairs of red marked edges do not span hypercubes, whereas the blue edges do:

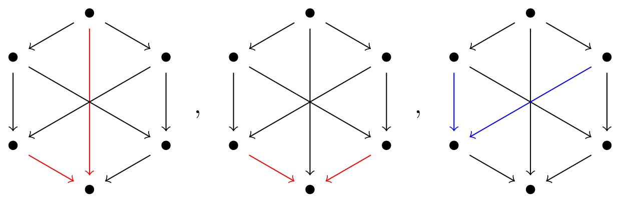

It should be clear that the first pair of red edges do not span a hypercube. In the second example, the issue is that there is not a *unique* way to map in a hypercube, with these specified base vertices.

We now come to a key definition. Suppose that E is a set of arrows with target x as above. We say that E *spans a hypercube cluster* if every subset $E' \subseteq E$ consisting of arrows with pairwise incomparable sources spans a hypercube.[5]

Example 2.4. Continuing the previous example, the pairs of blue arrows span hypercube clusters, whereas the red arrows do not:

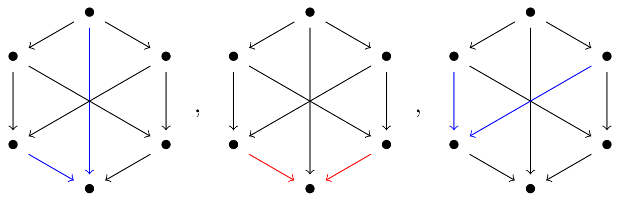

In the first diagram, the sources of the blue arrows are comparable in X, so the condition to span a hypercube cluster reduces to each singleton spanning a hypercube, which is trivially the case.

If E spans a hypercube, and $F \subset E$ is any subset of edges, then there is a maximal subset $F_{\text{max}} \subset F$ such that all arrows in F_{max} have incomparable sources. (The set F is a poset in a natural way, and F_{max} simply consists of its maximal elements.) Define the *hypercube map*

$$\theta : F \mapsto \text{crown of the hypercube spanned by } F_{\text{max}}.$$

2.3. **Diamonds.** Given a directed graph X, a *diamond* in X is a subgraph isomorphic to

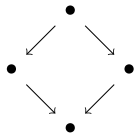

A full subgraph $J \subset X$ is *diamond complete*[6] if whenever it contains two edges sharing a node, it contains the entire diamond. In other words, for all diamonds in X, if J contains the red edges in any of the diagrams below, it necessarily contains the black edges as well:

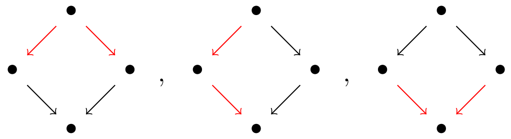

2.4. **Hypercube decompositions.** Recall that $X = [x, y]$ denotes the Bruhat graph of the interval between x and y. The following is the most important definition of this work. We say that a full subgraph $J \subset X$ is a *hypercube decomposition* if

(1) $J = \{v \in X \mid v \leqslant z\}$ for some $y \neq z \in X$, and J is diamond complete;
(2) for all $v \in J$, the set $E = \{\alpha : u \to v \mid u \notin J\}$ spans a hypercube cluster.

[5]Elements a and b in a poset are *incompable* if neither $a \leqslant b$ nor $b \leqslant a$ holds.
[6]This notion is due to Patimo [Pat21].

Example 2.5. Continuing Example 2.3, here are some possible choices of J (indicated by red):

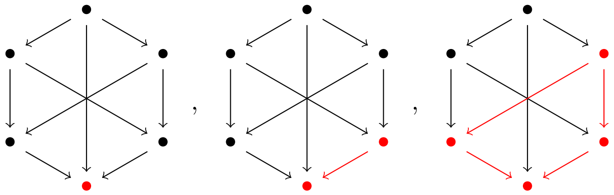

Only the middle choice of J constitutes a hypercube decomposition. In the example on the left, the edges arriving at the base vertex do not span a hypercube cluster. In the example on the right, J is not diamond complete. Here is the incomplete diamond:

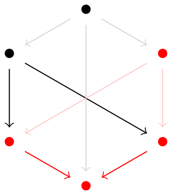

2.5. **The hypercube piece.** We are now ready to define the hypercube piece and inductive piece in our formula. We assume that X is the Bruhat graph corresponding to the interval $[x, y]$ and that we have fixed a hypercube decomposition $J \subset X$. The set

$$E = \{\alpha : v \to x \mid v \notin J\}$$

spans a hypercube cluster by definition. In particular we have a hypercube map:

$$\theta : \text{subsets of } E \to X$$

We consider the polynomial:

$$\widetilde{Q}_{x,y,J} = \sum_{\varnothing \neq I \subset E} (q-1)^{|I|-1} P_{\theta(I),y} \in \mathbb{Z}[q].$$

(Note that we may assume that all terms on the right hand side are known by induction.) We define the hypercube piece as follows:

$$Q_{x,y,J} = q^{\ell(y)-\ell(x)-1} \widetilde{Q}_{x,y,J}(q^{-1}).$$

2.6. **The inductive piece.** Let $J \subset X$ and consider the free $\mathbb{Z}[q]$-module:

$$M_J = \bigoplus_{x \neq v \in J} \mathbb{Z}[q] \delta_v.$$

This has a *standard basis* $\{\delta_v \mid v \in J\}$. If we define

$$b_v = \sum_{x \neq w \in J} P_{w,v} \cdot \delta_w$$

then $\{b_v \mid x \neq v \in J\}$ is also a basis for M, which we call the *Kazhdan-Lusztig basis*. This basis is known by induction.

We may now define the inductive piece. Recall that X is a Bruhat interval, with top node y. Define

$$r_{x,y,J} = \sum_{x \neq v \in J} P_{v,y} \cdot \delta_v \in M.$$

(In other words we consider all inductively computed Kazhdan-Lusztig and "restrict" to J.) Now expand r in the Kazhdan-Lusztig basis:

$$r_{x,y,J} = \sum_{x \neq v \in J} \gamma_v \cdot b_v.$$

The inductive piece is defined as follows:

$$I_{x,y,J} = \sum_{x \neq v \in J} \gamma_v \cdot \partial P_{x,v}.$$

2.7. **A theorem and a conjecture.** As above, $X = [x, y]$ denotes the Bruhat graph of the interval between x and y.

Suppose for a moment that we know the labelling of the nodes of X by permutations. (Note that this is forbidden information in the combinatorial invariance conjecture.) In this case, consider the full subgraph

$$L = \{v \in X \mid v^{-1}(0) = x^{-1}(0)\} \subset X.$$

Remark 2.6. For any node $v \in L$, the "hypercube edges" (i.e. those edges with targed v and source $\notin L$) are those edges corresponding to swapping 0 and i in v. These are precisely the edges which saliency analysis tell us are most important in our machine learning models (see Figure 3(a) in the main paper). This was our initial motivation for considering L.

We have:

Theorem 2.7. *$L \subset X$ is a hypercube decomposition, and we have:*

$$\partial P_{x,y} = I_{x,y,L} + Q_{x,y,L}.$$

This is a powerful new formula for Kazhdan-Lusztig polynomials for symmetric groups, and should have other applications. However, it does not solve the combinatorial invariance conjecture, as the node labellings are needed to define L.

Theorem 2.7 motivated us to consider more general hypercube decompositions. Remarkably, it seems that this combinatorial notion is exactly what is needed to make the above theorem hold:

Conjecture 2.8. *For any hypercube decomposition $J \subset X$ we have*

$$\partial P_{x,y} = I_{x,y,J} + Q_{x,y,J}.$$

Some remarks on this conjecture:

(1) We have just seen that any interval admits a hypercube decomposition, and thus the conjecture implies the combinatorial invariance conjecture for symmetric groups.
(2) The conjecture is equivalent to the statement that $I_{x,y,J} + Q_{x,y,J}$ is independent of the choice of hypercube decomposition.
(3) We have considerable computational evidence for this conjecture. It has been checked for all hypercube decompositions of all Bruhat intervals up to S_7, and over a million non-isomorphic intervals in S_8 and S_9.

Positivity plays an important role in Kazhdan-Lusztig theory. Remarkably, both pieces $I_{x,y,J}$ and $Q_{x,y,J}$ in our formula should have positive coefficients:

(1) The polynomials $Q_{x,y,J}$ have positive coefficients. (This can be shown directly, using the “unimodality of Kazhdan-Lusztig polynomials” [Irv88, BM01].)
(2) Conjecturally, the polynomials γ_v involved in the computation of the inductive piece have positive coefficients. This has also been checked in all the cases mentioned above, and is true for the hypercube decomposition L discussed above.

Remark 2.9. It is interesting to ask whether our formula might solve the combinatorial invariance conjecture for Coxeter groups other than symmetric goups. It does not, as one can see by inspecting the 5-crown:

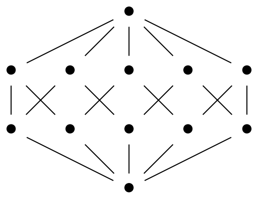

This occurs as a Bruhat graph of an interval in the group H_3 of symmetries of the icosahedron (see [BB05, §2.8]). One can check directly that it does not admit a hypercube decomposition.

2.8. **Two worked examples.** We give the reader two examples of our formula in action. These examples are illustrated in Figures 5 and 6.

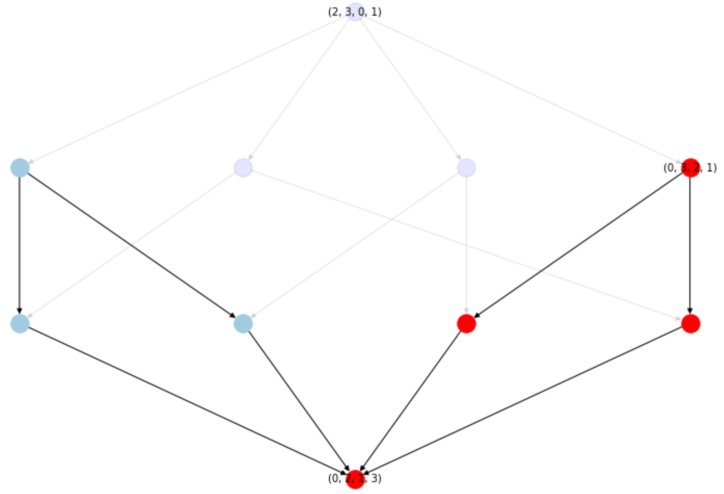

FIGURE 5. The Bruhat graph for the interval between $x = (0,2,1,3)$ and $y = (2,3,0,1)$. The image of the hypercube map at x is shaded blue, and the inductive piece is shaded red. All Kazhdan-Lusztig polynomials $P_{z,y}$ for $z \neq x$ are 1. All hypercube decompositions of this interval are isomorphic to this one.

2.8.1. *The interval between* $x = (0,2,1,3)$ *and* $y = (2,3,0,1)$. This is the first non-trivial example of a Kazhdan-Lusztig polynomial. In several respects this example is “too simple”, but we discuss it anyway. The interval together with a choice of hypercube decomposition is illustrated in Figure 5. (The reader may check that in this example all hypercube decompositions are isomorphic.)

We have
$$P_{x,y} = 1 + q \quad \text{and} \quad \partial P_{x,y} = 1 + 2q + q^2.$$
In this case the polynomial $\widetilde{Q}_{x,y,J}$ is
$$1 + 1 + (q - 1) = 1 + q$$
and hence the hypercube piece is
$$q^2(1 + q^{-1}) = q + q^2.$$
The inductive piece is
$$1 + q$$
and we indeed we have
$$\partial P_{x,y} = (1 + q) + (q + q^2).$$

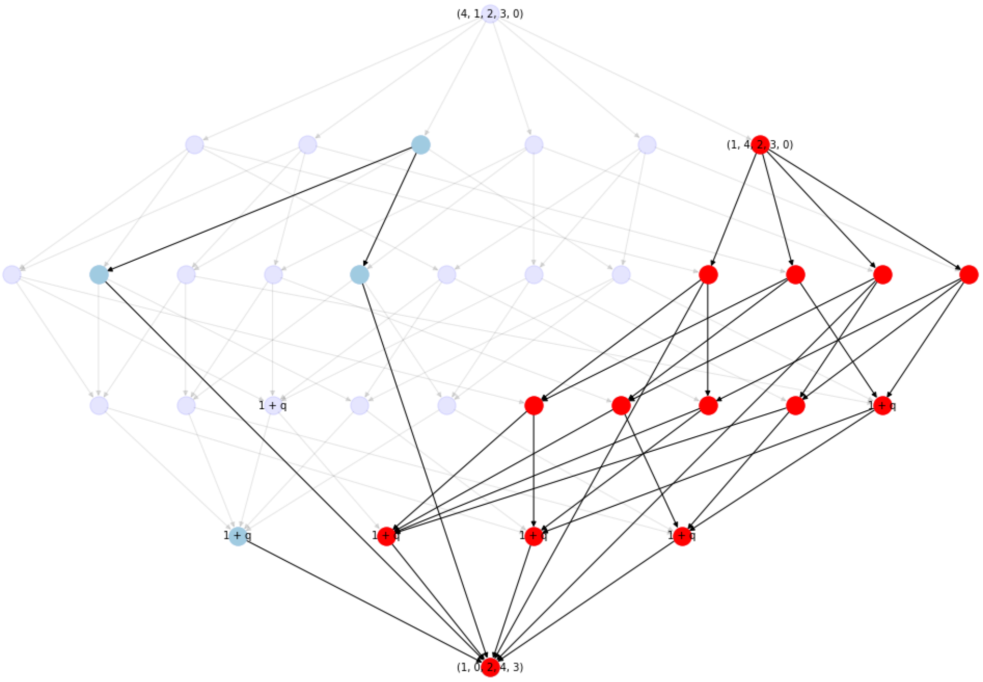

FIGURE 6. The Bruhat graph for the interval between $x = (1,0,2,4,3)$ and $y = (4,1,2,3,0)$ with a choice of hypercube decomposition. The image of the hypercube map at x is shaded blue, and the inductive piece is shaded red. All Kazhdan-Lusztig polynomials $P_{z,y}$ for $z \neq x$ are 1 unless indicated.

2.8.2. *The interval between $x = (1,0,2,4,3)$ and $y = (4,1,2,3,0)$.* This is a more interesting case, and illustrates several features of the general case. The interval together with a choice of hypercube decomposition J is illustrated in Figure 6.

The reader may check with a little work that we have

$$\widetilde{Q}_{x,y,J} = 1 + 2q + q^2$$

and hence

$$Q_{x,y,J} = q^4(1 + 2q^{-1} + q^{-2}) = q^2 + 2q^3 + q^4.$$

We now turn to the inductive piece. We have

$$r_{x,y,J} = b_{14230} + q \cdot b_{10432}.$$

(In Figure 6, 10432 is the only node of length $\ell(x) + 2$ in the inductive piece with non-trivial Kazhdan-Lusztig polynomial.) In particular,

$$\begin{aligned} I_{x,y,J} = \partial P_{x,14230} + q \cdot \partial P_{x,10432} &= 1 + 2q + 2q^2 + q^3 + q(1+q) \\ &= 1 + 3q + 3q^2 + q^3 \end{aligned}$$

and we deduce correctly that

$$\partial P_{x,y} = (1 + 3q + 3q^2 + q^3) + (q^2 + 2q^3 + q^4) = 1 + 3q + 4q^2 + 3q^2 + q^4.$$

Or in other words

$$P_{x,y} = 1 + 2q + q^2.$$

References

[BB05] A. Björner and F. Brenti. *Combinatorics of Coxeter groups*, volume 231 of *Graduate Texts in Mathematics*. Springer, New York, 2005. 1, 11

[BCM06] F. Brenti, F. Caselli, and M. Marietti. Special matchings and Kazhdan-Lusztig polynomials. *Adv. Math.*, 202(2):555–601, 2006. 4

[BM01] T. Braden and R. MacPherson. From moment graphs to intersection cohomology. *Math. Ann.*, 321(3):533–551, 2001. 11

[Bre04] F. Brenti. Kazhdan-Lusztig polynomials: history, problems, and combinatorial invariance. *Sém. Lothar. Combin.*, 49:Art. B49b, 30, 2002/04. 1, 4

[Car94] J. B. Carrell. The Bruhat graph of a Coxeter group, a conjecture of Deodhar, and rational smoothness of Schubert varieties. In *Algebraic groups and their generalizations: classical methods (University Park, PA, 1991)*, volume 56 of *Proc. Sympos. Pure Math.*, pages 53–61. Amer. Math. Soc., Providence, RI, 1994. 3

[Dye93] M. J. Dyer. The nil Hecke ring and Deodhar's conjecture on Bruhat intervals. *Invent. Math.*, 111(3):571–574, 1993. 3

[EMTW] B. Elias, S. Makisumi, U. Thiel, and G. Williamson. 1

[Hum90] J. E. Humphreys. *Reflection groups and Coxeter groups*, volume 29 of *Cambridge Studies in Advanced Mathematics*. Cambridge University Press, Cambridge, 1990. 1

[Irv88] R. S. Irving. The socle filtration of a Verma module. *Ann. Sci. École Norm. Sup. (4)*, 21(1):47–65, 1988. 11

[KL79] D. Kazhdan and G. Lusztig. Representations of Coxeter groups and Hecke algebras. *Invent. Math.*, 53(2):165–184, 1979. 1

[Pat21] L. Patimo. A combinatorial formula for the coefficient of q in Kazhdan-Lusztig polynomials. *Int. Math. Res. Not. IMRN*, (5):3203–3223, 2021. 8

[Soe97] W. Soergel. Kazhdan-Lusztig polynomials and a combinatoric[s] for tilting modules. *Represent. Theory*, 1:83–114 (electronic), 1997. 1
